# Supplementary material for: Development and evaluation of the psychometric properties of a brief parenting scale (PS-7) for the parents of adolescents
Source: PLoS One. 2020 Jan 29;15(1):e0228287. doi: 10.1371/journal.pone.0228287 (PMC6988928; doi:10.1371/journal.pone.0228287)
Supplement: S1 Dataset — (ZIP) [file pone.0228287.s001.zip › R syntax.docx]

PS-26

library(lavaan)

PS26 <- ' lax =~ PS_lax_1 + PS_lax_2 + PS_lax_3 + PS_lax_4 + PS_lax_5 + PS_lax_6 + PS_lax_7 + PS_lax_8 + PS_lax_9 + PS_lax_10 + PS_lax_11_PS_ver_2

ove =~ PS_over_1 + PS_over_2 + PS_over_3 + PS_over_4 + PS_over_5 + PS_over_6 + PS_over_7 + PS_over_8 + PS_over_9_PS_ver_5 + PS_over_10

ver =~ PS_ver_1 + PS_lax_11_PS_ver_2 + PS_ver_3 + PS_ver_4 + PS_over_9_PS_ver_5 + PS_ver_6 + PS_ver_7 '

fit <- cfa(PS26, data=Set1, order=c("PS_lax_1", "PS_lax_2", "PS_lax_3", "PS_lax_4", "PS_lax_5", "PS_lax_6", "PS_lax_7", "PS_lax_8", "PS_lax_9", "PS_lax_10", "PS_lax_11_PS_ver_2", "PS_over_1", "PS_over_2", "PS_over_3", "PS_over_4", "PS_over_5", "PS_over_6", "PS_over_7", "PS_over_8", "PS_over_9_PS_ver_5", "PS_over_10", "PS_ver_1", "PS_ver_3", "PS_ver_4", "PS_ver_6", "PS_ver_7"))

fitMeasures(fit)

fitMeasures(fit, "cfi")

fitMeasures(fit, c("chisq", "df", "pvalue", "cfi", "rmsea"))

summary(fit, fit.measures=TRUE, modindices=TRUE)

PS-21

library(lavaan)

PS21 <- ' lax =~ PS_lax_1 + PS_lax_2 + PS_lax_3 + PS_lax_4 + PS_lax_5 + PS_lax_6 + PS_lax_7 + PS_lax_8 + PS_lax_9 + PS_lax_10 + PS_lax_11_PS_ver_2

ove =~ PS_over_1 + PS_over_2 + PS_over_3 + PS_over_4 + PS_over_5 + PS_over_6 + PS_over_7 + PS_over_8 + PS_over_9_PS_ver_5 + PS_over_10 '

fit <- cfa(PS21, data=Set1, order= c("PS_lax_1", "PS_lax_2", "PS_lax_3", "PS_lax_4", "PS_lax_5", "PS_lax_6", "PS_lax_7", "PS_lax_8", "PS_lax_9", "PS_lax_10", "PS_lax_11_PS_ver_2", "PS_over_1", "PS_over_2", "PS_over_3", "PS_over_4", "PS_over_5", "PS_over_6", "PS_over_7", "PS_over_8", "PS_over_9_PS_ver_5", "PS_over_10"))

fitMeasures(fit)

fitMeasures(fit, "cfi")

fitMeasures(fit, c("chisq", "df", "pvalue", "cfi", "rmsea"))

summary(fit, fit.measures=TRUE, modindices=TRUE)

PS-13

library(lavaan)

PS13 <- ' lax =~ PS_lax_2 + PS_lax_3 + PS_lax_5 + PS_lax_6 + PS_lax_9

ove =~ PS_over_1 + PS_over_3 + PS_over_5 + PS_over_6 + PS_over_8

hos =~ PS_over_2 + PS_over_4 + PS_over_7 '

fit <- cfa(PS13, data=Set1, order=c("PS_lax_2", "PS_lax_3", "PS_lax_5", "PS_lax_6", "PS_lax_9", "PS_over_1", "PS_over_2", "PS_over_3", "PS_over_4", "PS_over_5", "PS_over_6", "PS_over_7", "PS_over_8"))

fitMeasures(fit)

fitMeasures(fit, "cfi")

fitMeasures(fit, c("chisq", "df", "pvalue", "cfi", "rmsea"))

summary(fit, fit.measures=TRUE, modindices=TRUE)

PS-12

library(lavaan)

PS12 <- ' lax =~ PS_lax_4 + PS_lax_2 + PS_lax_5 + PS_lax_1 + PS_lax_6 + PS_lax_3

ove =~ PS_over_6 + PS_over_4 + PS_over_5 + PS_over_2 + PS_over_3 + PS_over_1'

fit <- cfa(PS12, data=Set1, order=c("PS_lax_4", "PS_lax_2", "PS_lax_5", "PS_lax_1", "PS_lax_6", "PS_lax_3", "PS_over_6", "PS_over_4", "PS_over_5", "PS_over_2", "PS_over_3", "PS_over_1"))

fitMeasures(fit)

fitMeasures(fit, "cfi")

fitMeasures(fit, c("chisq", "df", "pvalue", "cfi", "rmsea"))

summary(fit, fit.measures=TRUE, modindices=TRUE)

PS-10

library(lavaan)

PS10 <- ' lax =~ PS_lax_9 + PS_lax_3 + PS_lax_5 + PS_lax_6 + PS_lax_2

ove =~ PS_over_1 + PS_over_3 + PS_over_6 + PS_over_8 + PS_over_5 '

fit <- cfa(PS10, data=Set1, order=c("PS_lax_9", "PS_lax_3", "PS_lax_5", "PS_lax_6", "PS_lax_2", "PS_over_1", "PS_over_3", "PS_over_6", "PS_over_8", "PS_over_5"))

fitMeasures(fit)

fitMeasures(fit, "cfi")

fitMeasures(fit, c("chisq", "df", "pvalue", "cfi", "rmsea"))

summary(fit, fit.measures=TRUE, modindices=TRUE)

PS-8

library(lavaan)

PS8 <- ' lax =~ PS_lax_10 + PS_lax_5 + PS_lax_4 + PS_lax_1

ove =~ PS_over_6 + PS_over_5 + PS_over_4 + PS_over_2 '

fit <- cfa(PS8, data=Set1, order=c("PS_lax_10", "PS_lax_5", "PS_lax_4", "PS_lax_1", "PS_over_6", "PS_over_5", "PS_over_4", "PS_over_2"))

fitMeasures(fit)

fitMeasures(fit, "cfi")

fitMeasures(fit, c("chisq", "df", "pvalue", "cfi", "rmsea"))

summary(fit, fit.measures=TRUE, modindices=TRUE)

PS-7

library(lavaan)

PS7 <- ' lax =~ PS_lax_4 + PS_lax_3 + PS_lax_2

ove =~ PS_over_3 + PS_over_6 + PS_over_8 + PS_over_5 '

fit <- cfa(PS7, data=Set1, order=c("PS_lax_4", "PS_lax_3", "PS_lax_2", "PS_over_3", "PS_over_6", "PS_over_8", "PS_over_5"))

fitMeasures(fit)

fitMeasures(fit, "cfi")

fitMeasures(fit, c("chisq", "df", "pvalue", "cfi", "rmsea"))

summary(fit, fit.measures=TRUE, modindices=TRUE)
