# Supplementary material for: Development and evaluation of the psychometric properties of a brief parenting scale (PS-7) for the parents of adolescents
Source: PLoS One. 2020 Jan 29;15(1):e0228287. doi: 10.1371/journal.pone.0228287 (PMC6988928; doi:10.1371/journal.pone.0228287)
Supplement: S1 Appendix — (DOCX) [file pone.0228287.s002.docx]

**Appendix. Factor structure of Parenting Scale and different shortened versions**

|  | PS-26 | PS-21 | PS-13 | PS-12 | PS-10 | PS-8 | PS-7 |
| --- | --- | --- | --- | --- | --- | --- | --- |
| **Laxness** |  |  |  |  |  |  |  |
| 7. I threaten to do things that I know I won’t actually do. | L, V | L | - | - | - | - | - |
| 8. I am the kind of parent that lets my child do whatever he/she wants. | L | L | - | - | - | L | - |
| 12. When I want my child to stop doing something, I coax or beg my child to stop. | L | L | L | - | L | - | - |
| 15. When we’re not at home, I let my child get away with a lot more. | L | L | - | - | - | - | - |
| 16. When my child does something I don’t like, I often let it go. | L | L | L | L | L | - | L |
| 19. When my child doesn’t do what I ask, I often let it go or end up doing it myself. | L | L | L | L | L | L | - |
| 20. When I give a fair threat or warning, I often don’t carry it out. | L | L | - | L | - | - | L |
| 21. If saying “No” doesn’t work, I offer my child something nice so he/she will behave. | L | L | L | L | L | L | - |
| 24. If my child misbehaves and then acts sorry, I let it go that time. | L | L | - | - | - | - | - |
| 26. When I say my child can’t do something, I let my child do it anyway. | L | L | - | L | - | L | - |
| 30. If my child gets upset when I say “No”, I back down and give in to my child. | L | L | L | L | L | - | L |
| **Overreactivity** |  |  |  |  |  |  |  |
| 3. When I’m upset or under stress, I am picky and on my child’s back. | O | O | O | O | O | - | - |
| 6. When my child misbehaves, I usually get into a long argument with my child. | O | O | O | O | O | - | O |
| 9. When my child misbehaves, I give my child a long lecture. | O, V | O | - | - | - | - | - |
| 10. When my child misbehaves, I raise my voice or yell. | O | O | O | O | O | O | O |
| 14. After there’s been a problem with my child, I often hold a grudge. | O | O | O | - | O | - | O |
| 17. When there’s a problem with my child, things build up and I do things I don’t mean to do. | O | O | O | O | O | O | O |
| 18. When my child misbehaves, I spank, slap, grab, or hit my child. | O | O | H | - | - | - | - |
| 22. When my child misbehaves, I get so frustrated or angry that my child can see I’m upset. | O | O | H | O | - | O | - |
| 25. When my child misbehaves, I almost always use bad language. | O | O | - | - | - | - | - |
| 28. When my child does something I don’t like, I insult my child, say mean things, or call my child names. | O | O | H | O | - | O | - |
| **Verbosity** |  |  |  |  |  |  |  |
| 2. Before I do something about a problem, I give my child several reminders or warnings. | V | - | - | - | - | - | - |
| 4. When I tell my child not to do something, I say a lot. | V | - | - | - | - | - | - |
| 11. If saying “No” doesn’t work right away, I keep talking and trying to get -through to my child. | V | - | - | - | - | - | - |
| 23. When my child misbehaves, I make my child tell me why he/she did it. | V | - | - | - | - | - | - |
| 29. If my child talks back or complains when I handle a problem, I give my child a talk about not complaining. | V | - | - | - | - | - | - |
| **Items not on a specific factor** |  |  |  |  |  |  |  |
| 1. When my child misbehaves, I do something about it later. | - | - | - | - | - | - | - |
| 5. When my child pesters me, I can’t ignore the pestering. | - | - | - | - | - | - | - |
| 13. When my child is out of my sight, I often don’t know what my child is doing. | - | - | - | M | - | - | - |
| 27. When I have to handle a problem, I tell my child I am sorry about it. | - | - | - | - | - | - | - |

Note: L = Laxness; O = Overreactivity; V = Verbosity; H = Hostile; M = Monitoring; PS-26 (15); PS-21 (20); PS-13 (30); PS-12 (35); PS-10 (31); PS-8 (16); PS-7 = This study.
