# Supplementary material for: Development and evaluation of the psychometric properties of a brief parenting scale (PS-7) for the parents of adolescents
Source: PLoS One. 2020 Jan 29;15(1):e0228287. doi: 10.1371/journal.pone.0228287 (PMC6988928; doi:10.1371/journal.pone.0228287)
Supplement: Attachment — Submitted filename: Response to Reviewers.docx [file pone.0228287.s003.docx]

Dear Editor,

Thank you for inviting us to resubmit the manuscript to PLOS ONE. In view of the comments from the reviewers, we have made substantial edits to the manuscript. Here we list the comments and the changes.

We have carefully considered all issues raised by the Editor, Reviewer 1 and Reviewer 2 and have made all edits that have been suggested. We appreciate your consideration of the revised manuscript.

Yours sincerely,

Dr. S. Fung

**Response to Reviewers**

| **Comments from Editor** | **Responses from Authors** |
| --- | --- |
| Both reviewers and I note that the conceptual foundation for the work was fairly modest. As the PS was developed with young children, more needs to be stated about how the measure translates to adolescence. | We have addressed this concern in the revised manuscript with additional discussion in the background section on the Parenting Scale to support the scale is applicable to both father and mother of early adolescent (p. 2). |
| Most critically, Reviewer 1 and I share the concern that no details are provided about how the short measure was developed. What criteria were employed to select the items? How many iterations of items were tested before arriving at the particular item set? The scientific processes that lead to the items and, ultimately, the measure is paramount. Without pre-registration, it becomes very challenging to identify serendipity in selecting the items, or a clear rational process. | We have responded it in the Reviewer 1 comment (point number 6). In addition, we also added the limitations and future research direction on p. 14. |
| Reviewer 1 also notes that additional statistical information is needed to evaluate the bifactor model | Additional EFA (with different estimators and rotation methods) was conducted with the 21-item version. The results did not support the bifactor structure. |
| Reviewer 2 noted that there was additional attention needed to fathers. I concur with the statement and press the issue further. In order to make stronger claims about fathers, additional analyses may be needed to clarify whether mothers and fathers provide similar or dissimilar responses. Moreover, it was not clear whether only one parent per household was included, or if there were some parent dyads who both completed the measures. If the latter, more attention in the analyses may be needed to accommodate non-independence of observations. | We also addressed this concern in Reviewer 2 (point number 2). We computed additional CFA on both mother and father samples. The results are identical to the samples consist of both parents (p. 11, Table 5).  There was only one parent per household was included in this study. |
| This is a fairly extensive set of comments to address. I cannot be sure that your work will ultimately be accepted for publication. However, there is interest in the work and the data come from an impressive sample. | Thank you for inviting us to resubmit the manuscript. We have carefully considered all issues raised by the Editor, Reviewer 1 and Reviewer 2 and have made all edits that have been suggested. |

| **Comments from Reviewer 1** | **Responses from Authors** |
| --- | --- |
| Reviewer #1: This manuscript focuses on assessing the psychometric properties of a brief, 7-item version of the parenting scale for use with parents of adolescents. Strengths of this paper include the assessment of psychometric properties of a parenting practices measure to be used with adolescents, as well as the utilization of a diverse population. Nevertheless, I do have several concerns. | Many thanks for your comments and feedbacks. We have made substantial edits to the manuscript to address your concerns. |
| 1.    There are several typos and misspellings. Please proofread carefully, as these errors make reading the manuscript difficult. Further, the manuscript appears to jump from topic to topic without transition, which makes reading the manuscript difficult. Please review the manuscript for overall flow. | The revised manuscript has been re-organised the structure and proofread carefully. |
| 2.    The explaining for why this scale should be used with parents of adolescents is not clearly outlined. Please provide a more thorough explanation, as the original measure was designed for use with parents of young children.  The manuscript was presented in a way that suggested the emphasis was on use with parents of adolescents; however, this is not discussed later within the manuscript. Please clarify what the primary goal of the manuscript is and why it is a significant contribution. | The Parenting Scale has been validated and used on parents of early adolescent. We have added the relevant literature in the introduction section (p. 2). |
| 3.    Additionally, there should be a more substantial focus in the Introduction on the use of the PS with Chinese families, and some of the details provided in the Discussion on cultural differences in parenting should be a focal point of the study rationale. | Agreed. In the revised manuscript, we added some relevant literature related to the Chinese parenting style (p. 2). |
| 4.    Please include the average age of the children in the study. Additionally, please provide more demographic information. | Our study only asked the parent’s age and other demographic information is presented in Table 1 (p. 4-5). |
| 5.    Please explain what a “complex item” is in more detail. As it currently stands, it is unclear precisely what this means or why it is referred to as “complex.” | Change to complicated, i.e. the item is related to more than one dimension/factor. |
| 6.    Please provide more detail regarding the procedure for creating the brief scale. How did the authors choose these seven items? They differ from other versions, so it is unclear how these items were selected. | In the revised manuscript, we have clarified the item selection procedure in p. 7.  In the discussion section (p. 13-14), we further illustrated that the brief scale with better psychometric properties and culturally universal, especially suitable for the Chinese context. |
| 7.    In the fourth paragraph of the procedures section, the Discussion regarding the PS and associations with parenting behaviors and child factors appears abruptly and should not be presented in this section. Please move to the rationale to the Introduction and detailed description of each measure in the measures section. | The discussion regarding the PS and associations with parenting behaviours and child factors in the methods section is to explain and justify the scales/variables for evaluating the criterion validity of the Parenting Scale. If move it to the introduction, it may affect the flow of the manuscript. But we do agree to provide detailed description of each measure in this section (p. 6). |
| 8.    At times throughout the manuscript, it is unclear if the authors are referring to the broader parenting scale literature, or the specific parenting scale they are validating. Please clarify. | The PS literature reviewed/quoted are all related to the specific parenting scale derived from Arnold et al. (1993). |
| 9.    It is unclear why it would be essential to reduce the scale from 21 items to 7. Additionally, it was stated that there is already an 8-item scale; therefore, is it necessary to reduce the scale by one item? Please explain. Further, research using IRT has shown that the 21-item version is superior to briefer versions (see Lorber et al., 2014). | The original Parenting Scale consist of 26 items with three factor structure. The 21-item version is a shortened version proposed by Salari et al. (2012) and evaluated by Lorber et al. in 2014. However, the existing Parenting Scale literature in recent years suggested that a briefer version is possessing better psychometric properties, such as the latest 8-item version.  This study also evaluated different versions with empirical data. Table 4 (p. 9) summarised the results of original scale (26 items with three factor structure), as well as the 21-item and other shortened versions. The CFA results show that none of them fulfilled the criteria for model fit. Hence, this study attempts to develop and validate a parenting style that can suitable the cultural context of the Chinese society.  Also, in the discussion section, some items are removed from the original scale mainly due to the concerns of the cultural/legal context in the Chinese society (p. 14). |
| 10. Given the sample size, the authors should test for measurement invariance across both parent and child gender. | We computed additional CFA analysis of the PS-7 on both father and mother respondents. The results also suggested that the proposed scale with good model fit (p. 11, Table 5). However, we did not collect the child gender, as this variable is usually not reported in other parenting scale studies. |
| 11. In the EFA there appeared to be high cross-loadings. With the 21-item version, could it be the case that a bifactor structure is more appropriate (e.g., lax, overactivity, and overall behavioral control)? | Additional EFA (with different estimators and rotation methods) was conducted with the 21-item version. The results did not support the bifactor structure. |
| 12. Please report both alpha and omega for reliability as well as 95% CIs for reliability (available in several R packages). | We have computed the alpha and omega for reliability with 95% CIs. Relevant texts are added in the methods and results sections (p. 6, 7 and 8). |
| 13. Lastly, a significant limitation of the current study is relying on only a single informant and not including test-retest reliably. | The sample was randomly stratified into three datasets based on the sex ratio of the respondents to avoid any potential biased of the analysis. We do agree that without test-retest reliability is a limitation of this study, we have added this in the discussion section (p. 14). |

**Reviewer 2**

| **Comments from Reviewer 2** | **Responses from Authors** |
| --- | --- |
| Reviewer #2: The submitted manuscript proposed an abbreviated version of the Parenting Scale (PS) for parents of adolescents based on a sample recruited from Hong Kong. This manuscript contributes to the parenting literature and measurement of parenting by indicating a shorter and more accessible form of an already well validated assessment of parenting. The authors used rigorous statistical and research methods and reported their findings clearly and concisely. This manuscript is a welcomed addition to the literature as it is an in-depth analysis of a parenting measure using an Asian sample, which is significant given the noted cultural differences in parenting practices with regard to paternalism as discussed by the authors. Further, I commend the author’s analysis of an existent parenting measure created narrowly for mothers of younger children and expanding these factors with a sample consisting of parents of adolescents including both mothers and fathers. | Thanks for the comments and feedbacks.  In the revised manuscript, we have provided additional discussion in the background section on the Parenting Scale to support the scale is applicable to both father and mother of early adolescent. We also took your advice to discuss the Asian parenting style (p. 2). |
| Along with the strengths of the manuscript discussed above, there were some areas of weakness that might strengthen its contribution to the literature. First, the authors discussed issues of validity as well as the problems with the statistical rigor of previous iterations of the PS in great detail but do not sufficiently state their theoretical basis for their aims and specific hypotheses based on said theory. The authors bring up many important variables that require further investigation in the measurement of parenting, such as lacking attention given to possible differences in parenting styles and behaviors with regard to child age (e.g. adolescents), historical failure to include diverse samples with regard to parent gender, as well as important cultural considerations when applying these measures globally. However, the manuscript would be improved by further discussion of hypotheses of how these items/factors may vary when studying only parents of adolescents. Similarly, consideration of how the inclusion of fathers may influence or improve these analyses and resultant proposed measure (e.g. do fathers parents differently, interaction of parent gender with the examination of adolescence and/or Asian culture). Discussion of differences in parenting in Asian cultures was addressed in the discussion section, but only to substantiate why items were removed from the outset. More thought and analysis of how these results fit into these multicultural concepts is warranted and an important addition to the literature. | We shared your concerns about these issues, the following are the changes we made it in the revised manuscript:  In the background section, we highlighted the importance of cultural considerations for the parenting scale (p. 2).  In the results section, we provided additional clarification of the development of the 7-item PS (p. 7).  We computed additional CFA on both mother and father samples. The results are identical to the samples consist of both parents (p. 11, Table 5).  In the discussion section, we proposed future study may consider incorporating diverse samples in different societies ideally with different parent gender and parent with different child age (p. 14). |

| **Comments from Editorial Office** | **Responses from Authors** |
| --- | --- |
| 1. We note that you have indicated that data from this study are available upon request. PLOS only allows data to be available upon request if there are legal or ethical restrictions on sharing data publicly. For more information on unacceptable data access restrictions, please see <http://journals.plos.org/plosone/s/data-availability#loc-unacceptable-data-access-restrictions>.   In your revised cover letter, please address the following prompts:  a) If there are ethical or legal restrictions on sharing a de-identified data set, please explain them in detail (e.g., data contain potentially sensitive information, data are owned by a third-party organization, etc.) and who has imposed them (e.g., an ethics committee). Please also provide contact information for a data access committee, ethics committee, or other institutional body to which data requests may be sent.  b) If there are no restrictions, please upload the minimal anonymized data set necessary to replicate your study findings as either Supporting Information files or to a stable, public repository and provide us with the relevant URLs, DOIs, or accession numbers. For a list of acceptable repositories, please see <http://journals.plos.org/plosone/s/data-availability#loc-recommended-repositories>.  We will update your Data Availability statement on your behalf to reflect the information you provide. | We have uploaded the data set as Supporting Information file through the online submission system. It can enables the future readers to replicate our findings. |
| 2. Please ensure that you refer to Figure 1 in your text as, if accepted, production will need this reference to link the reader to the figure. | Thank you for the reminder. We have added Figure 1 in the text (p. 9). |
| 3. Thank you for including your ethics statement on the online submission form:  "This study was approved by the ethical committee of the City University of Hong Kong. Its procedure was in compliance with the Declaration of Helsinki guidelines. All of the participants gave informed consent prior to the study.".  To help ensure that the wording of your manuscript is suitable for publication, would you please also add this statement at the beginning of the Methods section of your manuscript file. | We have added the ethics statement at the beginning of the Method section (p. 4). |
